# A Novel Method of Evaluating Knee Joint Stability of Patients with Knee Osteoarthritis: Multiscale Entropy Analysis with A Knee-Aiming Task

**DOI:** 10.1038/s41598-017-00411-5

**Published:** 2017-03-23

**Authors:** Diange Zhou, Shijie Zhang, Hui Zhang, Long Jiang, Jue Zhang, Jing Fang

**Affiliations:** 1 Arthritis Clinic & Research Center, Peking University People’s Hospital, Beijing, China; 2 Academy for Advanced Interdisciplinary Studies, Peking University, Beijing, China; 3 College of Engineering, Peking University, Beijing, China

## Abstract

Deteriorating knee stability is a local risk factor that reflects the occurrence and aggregative of osteoarthritis (OA). Despite the many biomechanics-based methods for assessing the structural stability of knee joints in clinics, these methods have many limitations. The stability of the knee joint relies on not only biomechanical factors, but also proprioception and the central nervous system. In this study, we attempt to depict the stability of knee joint from a holistic viewpoint, and a novel index of knee joint stability (IKJS) was thus extracted. We compared the differences of IKJS in 57 healthy volunteers and 55 patients with OA before and after total knee replacement (TKR). Analysis of Variance results demonstrated that there existed significant differences in IKJS among the three participating groups (<0.0001). Also, the IKJS of the operated leg in patients with knee OA increased remarkably after TKR (p < 0.0001). Furthermore, the results of the experiment suggested that the IKJS has sufficient reproducibility (ICC = 0.80). In conclusion, the proposed IKJS that employs the knee-aiming task is feasible for quantitatively determining knee stability. It can provide a potentially valuable and convenient tool to evaluate the effect of postoperative rehabilitation for patients with knee OA.

## Introduction

Many studies have realized that arthritic syndromes can significantly diminish the quality of life of patients^1^. Among those with osteoarthritis (OA), the most common pathogenic site is the knee. Compared with other sites, knee osteoarthritis (KOA) causes more local pain, thus restricting body movement and resulting in disability^2, 3^.

Instability of the knee joint can lead to minor injuries. The accumulation of these minor injuries can cause the occurrence and aggravation of knee osteoarthritis. Thus evaluation of dynamic joint stability of the knee is important to customized rehabilitation programs and may serve as an early warning sign of osteoarthritis.

There are two main methods to evaluate the stability of a knee joint. Biomechanics-based approaches, including the anterior drawer test, Lachman test, and pivot shift test, have been widely used to evaluate anterior cruciate ligament injuries when assessing the structural stability of knee joints in clinical settings. All of these methods are evaluated on the static structure of the knee joint. Another method is to check proprioception. The stability of the knee joint actually relies on not only biomechanical factors, such as muscle and ligament strength, but also proprioception, biomechanical factors, and the control of central nervous system. Unfortunately, there is no way to take into account these many factors in an overall evaluation from a macro point of view.

In fact, rehabilitation is a process that needs to be carried out continuously, even after a patient is discharged from the hospital.

Although the mechanism of maintaining the functionally stable knee is still not fully understood, a quantitative approach to evaluating functional knee stability is desirable for clinical use, especially for patients with knee OA, and is convenient for use at home. One promising approach is investigated starting with analysis of complex systems.

From the standpoint of complex systems is a promising way to analysis of biological phenomena^4–6^. Recently, the characteristic advantage of holism in assessing physiological signals have been increasingly acknowledged and valued^7, 8^. As a promising multiscale analysis approach, multi-scale entropy (MSE) has the unique capability to quantitatively extract the complexity of time series of physiological signals and has been used in analysis on signals of heart rate^9^, time series of gait^10^, and other physiological signals^11^. Since it was introduced by M. Costa^9^, MSE analysis has been successfully used to quantify the complexity of physiologic time series^9^ for 2002 years.

A study of Zhou^8^ designed an experimental procedure called standard aiming target test to quantitatively evaluate the effect of a vibrating insole on the specific task of aiming while standing. They found that the vibrating insole significantly improved the aiming performance and led to increased entropy in the fluctuations of aiming spot displacement.

Inspired by the complexity analyses of physiological signals under specific balance-maintaining tasks, we believed that if we can record the dynamic process of the knee joint in performing functional activities, we could evaluate it through a holistic methodology. Therefore, in this paper, we first proposed a knee-aiming task that could effectively reflect the stability of the knee joint. The complexity of the acquired dynamic pattern of aiming spot was then estimated using MSE, wherefore a novel index of knee joint stability (IKJS) was finally extracted.

Twelve healthy volunteers were recruited to evaluate the reproducibility of the IKJS. To further validate the effectiveness of the proposed strategy, we compared the differences in the IKJS among healthy volunteers, and patients with OA before and after total knee replacement (TKR) surgery. The ANOVA analysis was used to compare groups of quantitative variables. In addition, the paired t-test was used to examine the difference in IKJS of the operated leg in patients with OA before and after surgery.

## Method

### Subjects

These studies were approved by institutional review committees while assurances were approved by the Biomed-X Center of Peking University. Informed consent was required for participation. All methods were performed in accordance with the relevant ethical guidelines and regulations by including a statement in this section.

Fifty-seven healthy volunteers, including 2 males and 55 females (aged 42–70, averaged age 58.5 ± 6.1), were recruited as the healthy group. Any volunteer who complained of knee discomfort during walking in horizontal and vertical directions was excluded of this study.

In addition, we recruited 153 patients from the Department of Orthopedics, People’s Hospital Affiliated to Peking University between March 2014 to Oct 2014 as the operative group. All of the 153 patients, including 26 males and 127 females, were going to receive knee arthroplasty (age 53–93, averaged 68.8 ± 7.1 years). Two weeks after total knee replacement operation, 55 patients participated in the experiment (aged 53–83, averaged 69.4 ± 5.7 years). The inclusion criteria were:

(a) They have been diagnosed with KOA according to the original Kellgren and Lawrence (K&L) scale (grades 0–4, with 0 being normal and 4 severe OA). These patients were suitable for knee arthroplasty. (b) No muscle weakness was observed. (c) No vision impairment was observed. (d) The patient had a better range of motion with active adduction of 60/0/30°. (e) Patients recovered well after operations without thrombus. No movement disorder symptoms were noted for any of the patients. (f) Visual analogue scale (VAS) pain score less than 5.

### Knee-Aiming Task

**Figure 1:**
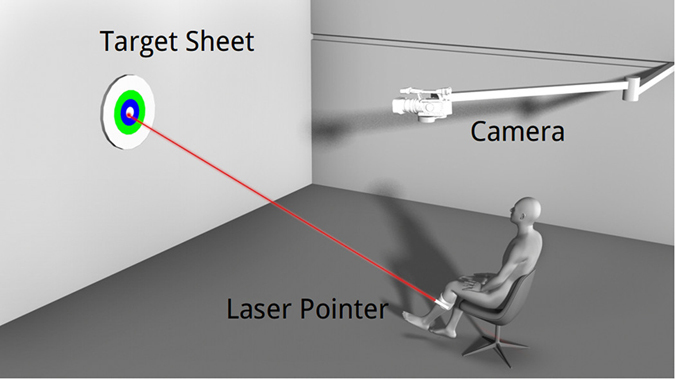
Schematic diagram of the knee-aiming task.participant sit on a chair or a bad with a Knee-Aiming Task.

All group of participants were asked to joined Knee-Aiming Task. Most healthy volunteers complete Knee-Aiming Task one time. Twelve of healthy volunteers complete Knee-Aiming Task once a day, a total of seven days for the repetitive test of out method. 153 patients complete Knee-Aiming Task before surgery. 55 patients complete Knee-Aiming Task in two weeks after total knee replacement operation. 27 patients of them have complete Knee-Aiming Task two times: before and after TKR. As shown in Fig. 1, a Knee-Aiming Task that could effectively reflect the stability of the knee joint was proposed in this study. The process of the knee-aiming task was performed as follows: (a) Participant was seated on the bed with his/her calves hanging off the edge of the bed with a knee-to-edge distance of 5–10 cm. (b) A laser pointer was fixed to the lateral calf of the leg with an adjustable strap. The angle of the laser pointer could be manually adjusted. (c) Participant sits 5 meters away from the target with his knees bent at a 60-degree angle with the floor. (d) Participant was required to focus and keep aiming at the preset target by using the laser pointer on his leg for 60 seconds. The moving light track on the target sheet was captured with a sampling rate of 100 frames per second by a high-definition camera (camputar ZN-C2M).

**Figure 2:**
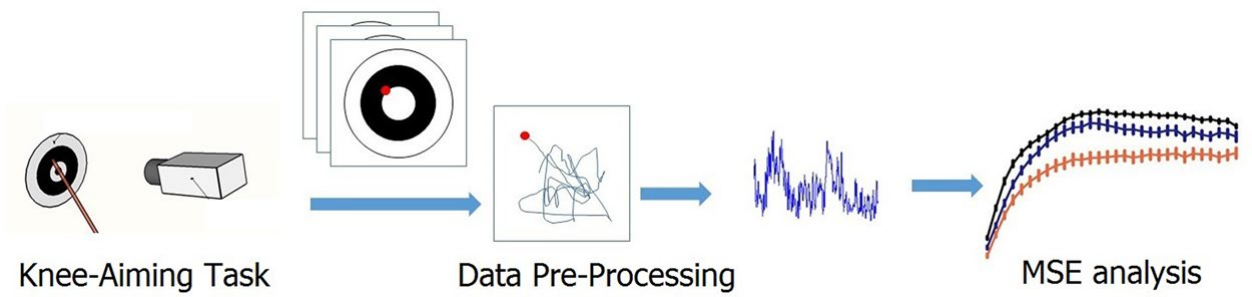
The flowchart of data analysis. Data gathered from the experiment first through a series of Data Pre-Processing. Then the MSE approach was further utilized to extract a novel biometrics-termed index of knee joint stability (IKJS) from the displacement time series in order to evaluate knee stability.

Data Pre-Processing. As shown in Fig. 2, for the data analysis, the magnitude series was used instead of the raw data to determine aiming spot data. The aiming spot time series recorded on the target sheet was obtained from a series of raw trace pictures. After using a grayscale image segmentation technique to exact the locations on the sampling picture, the absolute displacement of each sampling point was calculated by comparing with the previous one through the following:

1 $${\rm{d}}=\sqrt{{({x}_{i}-{x}_{i-1})}^{2}+{({y}_{i}-{y}_{i-1})}^{2}}$$

where d is the absolute displacement, and x (i) and y (i) are the coordinate values of current sampling points while x(i − 1) and y(i − 1) stand for the coordinates of the previous points^8^.

The Empirical Mode Decomposition (EMD) is used to smooth the displacement time series of the track of the moving light spot.

Compared with wavelet and fourier analysis, the EMD method has the advantage of using a fully adaptive basis derived from each data set by means of a sifting process^12^. EMD is a technique that adaptively decomposes a time series X(t) as a sum of simpler signs plus a residual according to the equation:

2 $$x(t)={\sum }_{i+1}^{n}{\psi }_{i}(t)+rn(t)$$

Where Ψi(t) is the simpler signs, and rn(t) is the residual.

We typically obtained 10 components with characteristic frequencies given by sample frequency/(2n + 1), where 1 ≤ n ≤ 10. The 10 IMFs have predominant power for frequencies ranging from approximately 0.1 to 50 Hz.

### MSE analysis

In our attempt, the MSE approach was further utilized to extract a novel biometrics-termed index of knee joint stability (IKJS) from the displacement time series in order to evaluate knee stability as illustrated in Fig. 2. Given one-dimensional discrete time course, the consecutive coarse-grained time series ${y}_{j}^{\tau }$ is constructed, we can write the equation as below:

3 $${y}_{j}^{\tau }=\frac{1}{\tau }{\sum }_{i=(j-1)\tau +1}^{j\tau }{x}_{i},1\le j\le \frac{N}{\tau }$$

In the equation, *x*_*i*_ stands for the original time series, τ represents the scale factor. The coarse-grained series at each scale τ are obtained by taking arithmetic average of τ which neighbors original values in non-overlapping windows. The length of the coarse-grained courses can be represented as $\frac{N}{{\tau }^{8}}$.

Then, the sample entropy (SE) is calculated for each coarse-grained time series ${y}_{j}^{\tau }$ and the group means are regarded as a function of each scale factor when the results are plotted. SE is an improved version of traditionally approximate entropy (AE), which measures the irregularity of a system, as calculated in the following steps:

(a) For the coarse-gained course of time scale form the vectors $\{Y(i)=y(i),y(i+1),\ldots ,y(i+{\rm{m}}\,-\,1)\}$, where m means the pattern length parameter; (b) Define the distance between vector Y(i) and Y(j) as max $(|y(i+k)-y(i-k)|)$; (c) For each $i\le N-m$, calculate the quality, ${B}_{j}^{m}$ (number of vectors $y(i),i\ne j$, such that $d(Y(i),Y(j)) < r$); (d) Repeat steps (1–3) with embedding dimension m + 1;

4 $$(e)\,{\rm{SE}}\,{\rm{is}}\,{\rm{defined}}\,{\rm{as}}:{S}_{E}=\mathop{\mathrm{lim}}\limits_{N\to \infty }\,\mathrm{ln}(\frac{{\sum }_{i=1}^{N-m}{B}_{j}^{m}}{{\sum }_{i=1}^{N-m}{B}_{i}^{m+1}})$$

According to previous reports, we chose a tolerance level of r = 0.15 × standard deviation (S.D.) of the time series to avoid distortion of SE values by variability in signal magnitude in addition to usual choice of the pattern length parameter m = 2. In this analysis, we computed the SE values for scales τ up to 30.

In this study, the IKJS was defined as the mean MSE values scale, ranging from 12 to 15.

### Statistical analysis

IKJS data are divided into three groups: healthy volunteers, preoperative patients, and postoperative patients. The analysis of variance (ANOVA) was employed to observe whether or not there existed significant differences among the three groups.In addition, the paired t-test was used to provide statistical relevance of the IKJS differences in 27 patients with OA before and after TKR.

## Result

### repeatability test

We compared the repeated measurement data in 12 healthy volunteers based on the IJKS. The intraclass correlation coefficient results for IJKS were 0.80 (95% CI = 0.49–0.94). This result demonstrates that the proposed method has a satisfying reproducibility.

### Comparative analysis of three groups

**Figure 3:**
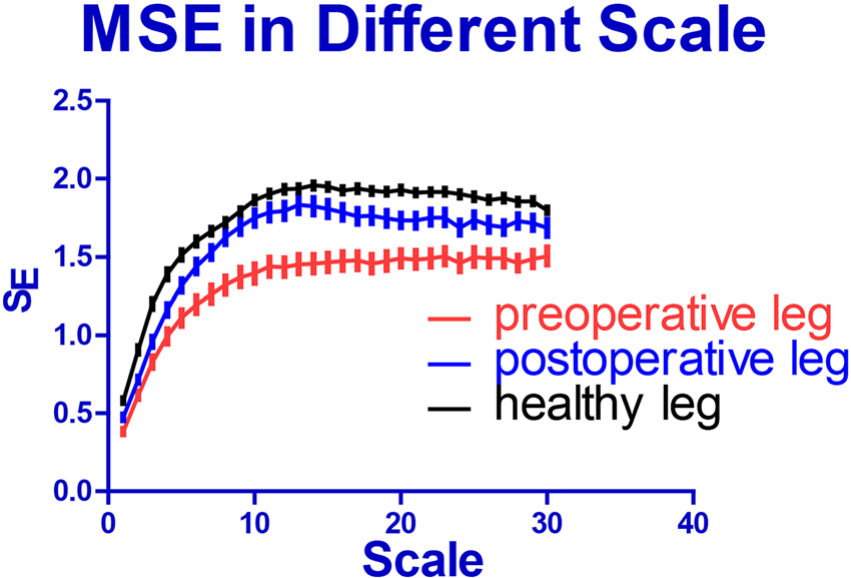
On the 1~30 scale of the MSE, a comparison between three groups. The MSE of displacement time series at scales 12–15 was observed to be different among three groups.

**Figure 4:**
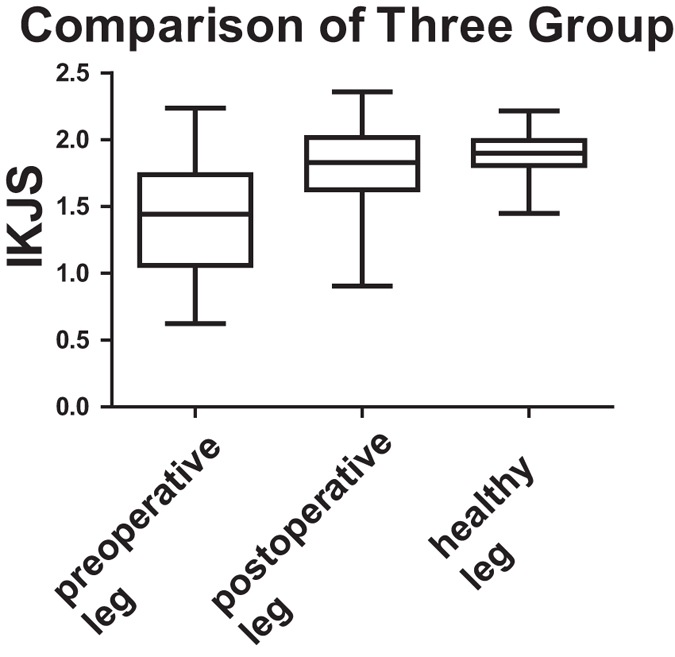
Comparisons between IKJS results of three groups. IKJS of healthy volunteers is better than patient, and postoperative patient comes closer to the healthy volunteers.

Figure 3 demonstrates comparisons between MSE results of the three groups. The IKJS was defined as the mean of the MSE values scale, ranging from 12 to 15. Compared with the leg before the surgery, the leg after surgery has a higher knee stability and got closer to the knee stability of healthy elderly people. Furthermore, ANOVA results suggest that there exist significant differences in IKJS among the three groups (p < 0.0001, Fig. 4).

### A compare with pre-and post operation

**Figure 5:**
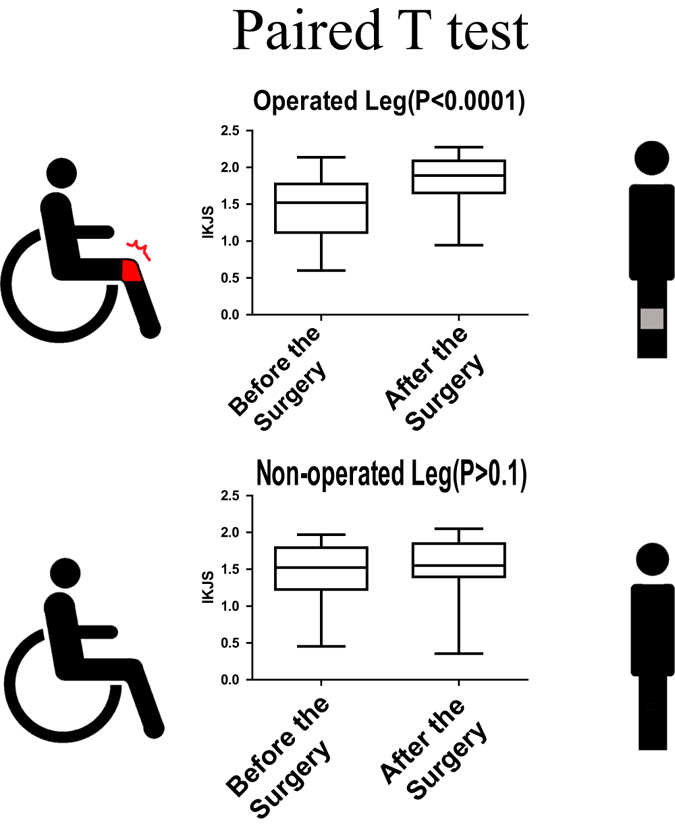
Paired t-test results of IJKS. (**A**) For operated leg pre/post-surgery. (**B**) For non-operated leg pre/post-surgery. The IKJS of operated leg has a significant improvement while non-operated leg has no significant change.

As shown in Fig. 5, the paired t-test showed significant difference (p < 0.001) between the mean scores from the two testing sessions. Meanwhile, the paired t-test comparison did not show a significant difference (p = 17) between non-operated leg before and after surgery (Figure B).

## Discussion

In this paper, we put forward a new index based on MSE to effectively evaluate the stability of the knee joint used in postoperative rehabilitation. The task design will obviously affect the method quality^12, 13^. The more difficult the balance task, the better reflection of the real situation^14, 15^. Therefore, we put forward a Knee-Aiming Task. This task is appropriate for patients and normal non-patients alike.

With the proposed knee-aiming task, the complex postural control process of the knee joint was measured by tracking a moving light spot. In the task, a laser pointer was fixed to the lateral calf of leg and the participant was seated on the bed with his/her calf hanging off. Therefore, only the movement of the knee could affect the aiming performance. Basically, functional knee stability is dynamic and based on adaptive capacity. In this study, we attempted to utilize adaptability of knee joint to reflect the functional knee stability. During the experiment, the subjects were required to focus and keep aiming at the target sheet. This process required patients to constantly correct knee movement, which effectively revealed the dynamic adaptability of their knee joint capacities.

Further, the EMD is used to smooth the displacement time series of the moving track of the light spot. Our study is limited to the analysis of temporal structures with characteristic scales ranging from 0.01 s (100 Hz) to 2 s (0.5 Hz); the removal of frequencies below 0.1 Hz assures that no relevant information is lost with the detrending process. Then, we used the MSE method to analyze the dynamic patterns in moving spot tracks. Actually, many studies have realized that MSE in physiological signals can exhibit the adaptability of dynamic control systems; the decreased MSE is associated with decreased adaptability. Therefore, IKJS extracted from MSE results was finally used to evaluate the stability of knee joints in this study.

The stability of the knee joint is a complex biomechanical process. We believe that dynamic patterns of aiming can amplify micro-physiological information, unaffected by subjective factors. Thus, it is easy to understand why the IKJS index has acceptable reproducibility, which has been confirmed by the results of ICC analysis.

By comparing MSE results, we found that OA patients after surgery have significantly higher IKJS than OA patients before surgery, which is in accordance with common sense and clinical experience.

As shown in Fig. 3, all subjects had positive trends in both MSE values on the scale from 1 to 12 (scale = 1:P < 0.4, scale = 12:p < 0.001). The reason for increasing group differences and entropy might have resulted from coarse graining procedure, which neglects the influence of the regular sports of leg. Actually, the random behavior can reduce the entropy value and will cover the micro regulation of receptor^11^. The frequency of scale 12 was about 8 Hz (0.125 s), while human reaction speeds were between 0.1 and 0.5 s. We considered that scale 12 corresponded to reaction speed of the human.

In scales between 15 and 30, the entropy of each group of subjects tends to be stable but slight fluctuating. This is probably due to the coarse graining procedure filtering out the noise from the signal. And the filter performance of the large scale is lower than that of the smaller scale^16^. Therefore, in our attempt, the scale ranging from 12 to 15 was selected to so IKJS would reflect stability of knee joint.

In addition, we realized that a long period of performing the aiming task could cause fatigue and thus lead to negative influence on the reproducibility of IKJS. In the current experiment design, the aiming duration was set at 60 s.

In conclusion, the proposed IKJS with knee-aiming task is feasible in quantitatively depicting knee stability. In addition, our method has good repeatability. It could provide a valuable and convenient tool to evaluate the effects of postoperative rehabilitation for patients with knee OA.
